# Active matrix metalloproteinase‐8: A potential biomarker of oral systemic link

**DOI:** 10.1002/cre2.516

**Published:** 2021-11-19

**Authors:** Kehinde Umeizudike, Ismo Räisänen, Shipra Gupta, Solomon Nwhator, Andreas Grigoriadis, Dimitra Sakellari, Timo Sorsa

**Affiliations:** ^1^ Department of Preventive Dentistry, Faculty of Dental Sciences College of Medicine, University of Lagos & Lagos University Teaching Hospital Lagos Nigeria; ^2^ Department of Oral and Maxillofacial Diseases Head and Neck Center, University of Helsinki and Helsinki University Hospital Helsinki Finland; ^3^ Oral Health Sciences Centre Post Graduate Institute of Medical Education & Research Chandigarh India; ^4^ Department of Preventive and Community Dentistry, Faculty of Dentistry College of Health Sciences, Obafemi Awolowo University Ile‐Ife Nigeria; ^5^ Department of Preventive Dentistry, Periodontology and Implant Biology Dental School, Aristotle University of Thessaloniki Thessaloniki Greece; ^6^ Division of Periodontology, Department of Dental Medicine Karolinska Institutet Stockholm Sweden

**Keywords:** aMMP‐8, biomarkers, clinical studies, diseases, health, matrix metalloproteinases, oral, periodontitis, systemic

## Abstract

**Objectives:**

This mini review aims to address some possible gaps in periodontal diagnosis in clinical studies particularly involving the oral‐systemic connection with a view to minimize such gaps, and thus improve patient treatment experiences and outcomes.

**Methods:**

The conventional assessment of periodontitis has traditionally been by clinical and radiographic oral parameters. We reviewed numerous studies published mainly within the past decade, to affirm the oral‐systemic link, the contribution of periodontitis to the inflammatory burden in various systemic diseases and conditions, and the potential role of active matrix metalloproteinase‐8 (aMMP‐8).

**Results:**

While it is established that periodontal pathogens in dental plaque biofilm are the primary initiating agents in periodontitis, it has become clear from the appraisal of recent studies that the host inflammation, including biomarkers such as aMMP‐8 play a major role, being the driving underlying pathological mechanism in both periodontitis and systemic diseases.

**Conclusions:**

The apparent limitations of conventional diagnostic tools have led researchers to seek alternative methods of evaluation such as the quantification of biomarkers including aMMP‐8, which can be a bridge between oral/periodontal and systemic diseases; aMMP‐8 can form a mouth‐body connection.

## 1 INTRODUCTION

The oral‐systemic link has been well established through a wide array of scientific literature, particularly from studies on periodontal‐systemic diseases. (Alpert, 2017; Beck et al., 2019; Genco & Sanz, 2020) Plaque biofilm levels, which reflect the oral hygiene are important triggers for oral inflammation and the ensuing systemic bacteremia, these form one of the proposed pathways in the oral‐systemic link. Periodontopathogens in dental biofilm are thought to constitute the “trigger” for the inflammatory/collagenolytic response that characterizes destructive periodontitis, (Golub & Lee, 2020) and are implicated in the pathogenesis of atherosclerosis. (Reyes et al., 2013) More recently, oral dysbiosis has been implied as the major mechanism in the pathogenicity of subgingival plaque, yet, its relationship with periodontal inflammation is temporal and the evidence of dysbiosis preceding destructive inflammation appears to be inconclusive. (Van Dyke et al., 2020; Van Dyke & Sima, 2020; Yost et al., 2015) The shift from infection to inflammation in the etiopathogenetic basis of periodontitis, (Van Dyke et al., 2020; Van Dyke & Sima, 2020) may impact on our understanding of the oral‐systemic link. Porphyromonas gingivalis, one of the most common pathogens in periodontitis‐related inflammatory disease, produces virulence factors like lipopolysaccharide (LPS) that can trigger an inflammatory cascade involving proinflammatory cytokines and matrix metalloproteinases (MMP). (Kuula et al., 2009) MMP‐8 (collagenase 2), a collagenolytic enzyme plays a significant role in tissue destruction in periodontitis as it instigates the digestion of type I collagen, which is the most dominant interstitial type in the periodontal tissues. (Kuula et al., 2009; Sorsa et al., 2016) Furthermore, elevated MMP‐8 levels have been demonstrated in many diseases such as atherosclerosis, inflammatory bowel disease, asthma, and oral cancer. (Kuula et al., 2009) Paradoxically, apart from its destructive effects on the tissues, MMP‐8 may exert anti‐inflammatory activity in the host defense by processing chemokines and cytokines that are anti‐inflammatory in nature. (Kuula et al., 2009; Sorsa et al., 2016)

Could there be some methodological gaps in our understanding of the basic pathogenesis of periodontitis which still accounts for the persistent high prevalence of 11.2% for its most destructive form, (Kassebaum et al., 2014) and its relationship with systemic diseases and why its precursor, gingivitis remains stable in some people, but progresses to periodontitis in others. To date, this has remained a dilemma (Van Dyke et al., 2020; Van Dyke & Sima, 2020) besides being ascribed to some unique host immune characteristics.

A recently published article, (Raittio & Farmer, 2021) titled the “Methodological gaps in studying the oral‐systemic disease connection,” highlights common gaps observed in epidemiological studies on the oral‐systemic link. The authors particularly emphasized the need for such studies to take into account the numerous individual and environmental variables that could form a complex nexus of common risk factors and pose a methodological challenge when explaining/interpreting such relationships. (Raittio & Farmer, 2021) Some recent studies (Pussinen et al., 2019; Pussinen et al., 2020) also stress upon the associations of childhood oral infections with cardiovascular risk factors and metabolic syndrome in adulthood respectively. These epidemiological studies are longitudinal cohorts by design and are very unique in that they elucidated the link between oral infection/inflammation in childhood and subclinical atherosclerosis and metabolic parameters in adults. It is noteworthy and commendable that the researchers were able to follow‐up the children participants for a long period of 21–31 years till adulthood. This appears to be among the first long‐term childhood oral‐systemic link studies with valuable contribution to our knowledge about the oral‐systemic relationship. (Pussinen et al., 2019; Pussinen et al., 2020) We do however share some of the concerns raised in the recently published short review, (Raittio & Farmer, 2021) and taking a cue from their article, we wish to address some aspects in this concise review in order to minimize the possible gaps in the oral–systemic connection, and thus improve patient treatment experience and outcomes.

## 2 APPRAISAL OF CAVEATS IN LITERATURE

Some studies (Pussinen et al., 2019; Pussinen et al., 2020) have utilized only clinical oral parameters to quantify oral infection/inflammation during childhood to assess the adult systemic effects, while controlling for other potential confounding factors such as high‐blood pressure and body mass index. Although, the authors found significant associations between childhood oral infections and increased carotid artery intima media thickness, metabolic parameters and metabolic syndrome in adulthood, we opine that the inclusion of an additional diagnostic tool; the pro‐inflammatory oral fluid biomarker such as active matrix metalloproteinase‐8 (aMMP‐8) to the clinical oral parameters in these longitudinal studies would have added more credence to the interpretation of the study outcomes, further strengthening their conclusions and making it more robust.

A longitudinal study (Lee et al., 1995) was one of the first to show in vivo, utilizing gingival crevicular fluid samples, the direct role of active MMP‐8, but not latent or total MMP‐8 (tMMP‐8), in the progression of periodontitis. Recent studies have demonstrated that aMMP‐8 is a more accurate biomarker of periodontitis compared to tMMP‐8. (Gupta, Sahni, et al., 2021; Hernández et al., 2021) To buttress this point, the recent review (Raittio & Farmer, 2021) pointed out the need for cautious interpretations when conducting epidemiological studies involving causal or predictive relationships especially when explanatory variables between exposures and outcomes are missing. The study of the oral‐health metabolic syndrome link may have been quick to conclude on the influence of oral inflammation or infection on the progression of hypertension, while stressing the demand for oral hygiene to prevent hypertension starting early in life. (Pussinen et al., 2019; Pussinen et al., 2020) The authors rightfully admitted some limitations of their studies, which included potential confounding factors such as the lack of microbiological profiling, dietary, or exercise habits; and the non‐assessment of the clinical attachment levels nor radiographic records, as well as use of a partial mouth recording index as only six teeth were examined. (Pussinen et al., 2019; Pussinen et al., 2020) The authors' definition of periodontal disease as gingival bleeding on probing and periodontal probing pocket depths is somewhat ambiguous and does not clearly distinguish between the different categories of periodontal health and disease. It is especially important to consider these factors in the light of the latest staging and grading of periodontitis that has been recommended. (Tonetti et al., 2018)

Even though, the adoption of this recent classification in both studies (Pussinen et al., 2019; Pussinen et al., 2020) might have proven to be challenging since the guidelines were only recently published, we still strongly feel that using the clear staging and grading system (Tonetti et al., 2018) would provide a better interpretation of the association between periodontal disease and developing subclinical atherosclerosis, metabolic syndrome or other systemic comorbidities. Undoubtedly, probing few index teeth alone for periodontal pockets may be convenient for children, but the value of performing a more comprehensive full mouth periodontal probing as adulthood sets in, is unrivaled in such a rigorous study. It is very possible that the partial records might have excluded some deep periodontal pockets and affected the final results. In this regard, utilizing a simple, noninvasive assay for a biomarker such as mouthrinse aMMP‐8 would have been an invaluable adjunctive measure to periodontal pocketing of the index teeth and possibly would have helped to detect and predict more accurately the periodontal attachment tissue loss in the ensuing years of the study. (Gul et al., 2020; Leppilahti et al., 2015; Pussinen et al., 2020)

## 3 THE EVOLUTION OF PERIODONTAL DIAGNOSIS

Current evidence correctly showcases the limitations of traditional practices of using clinical indices and radiographic parameters to evaluate the oral/periodontal diseases when used in isolation as they mostly reflect only past periodontal tissue destruction. (Kinane et al., 2017)

Besides being time consuming, they are often cumbersome, uncomfortable for patients, especially children and rely to a large extent on the clinician's skill, which may have some element of subjectivity. This point has been laid to rest in the latest case definition of periodontitis, which makes an effort to bridge this apparent gap in periodontal diagnosis by including serum and oral fluid biomarkers to improve diagnostic accuracy in the early detection of the risk of periodontal breakdown. (Tonetti et al., 2018) This is most vital in susceptible individuals when measuring periodontal disease or predicting the risk of future progression. (Tonetti et al., 2018) Good and invaluable as clinical oral parameters have been for decades in assessing periodontal disease by the diligent efforts of renowned epidemiologists and clinicians, these clinical parameters have imperfections being man‐designed/artificial‐made and are restrictive.

For instance, bleeding on probing (BOP), although a very useful clinical marker of active periodontal tissue inflammation, still has a low positive predictive value of ≤30% for attachment loss following repeated BOP. (Lang & Bartold, 2018) On the other hand, the absence of BOP has a high negative predictive value of 98%–99% in determining periodontal health/stability. (Lang & Bartold, 2018) Lately, a stronger association was found between aMMP‐8 and subclinical periodontitis compared to BOP test affecting 20% of sites and the aMMP‐8 point‐of‐care (POC) test had ≥ twice higher sensitivity than BOP. (Räisänen et al., 2019) The aMMP‐8 biomarker was therefore deemed as being more precise and effective than conventional BOP in its ability to identify subclinical periodontitis/pre‐periodontitis in adolescents. This thus reduces the risk of undertreatment. (Räisänen et al., 2019) Although the small sample size of the study participants was a limiting factor in the study, it can be conclusively said that the time has come for clinical oral parameters to be complemented in our everyday clinical practice with well researched and validated biomarkers (serum, gingival crevicular fluid [GCF], saliva, mouthrinses, peri‐implant sulcular fluid [PISF]) which accurately reflect/mimic the human biological processes. Such biomarkers, upon due validation could not only be mere adjuncts to periodontal diagnosis, but in certain settings, prove to be useful as part of point of care diagnostics in order to aid medical professionals and even laymen seeking referral or dental care.

## 4 AMMP‐8 AND THE NEW CLASSIFICATION OF PERIODONTITIS

Matrix metalloproteinase‐8 (MMP‐8) collagenase‐2/neutrophil collagenase is among the most recorded and satisfactory candidates for distinguishing between periodontal health and disease. (Herr et al., 2007; Nwhator et al., 2014; Sorsa et al., 1990; Sorsa et al., 2016; Sorsa et al., 2020) It is the major host‐derived collagenase that can degrade type I collagen of the periodontal supporting tissues and plays a role in both physiological activity and pathologic tissue destruction. (Herr et al., 2007; Nwhator et al., 2014; Sorsa et al., 1990; Sorsa et al., 2016; Sorsa et al., 2020) MMP‐8 exists in both inactive and active forms. Its active form in particular has been demonstrated to have (i) a stronger association with the periodontal status and (ii) a better diagnostic accuracy for periodontal disease than its total or latent forms. (Gul et al., 2020; Lee et al., 1995; Sorsa et al., 2016) More recently, an oral rinse POC immunoassay mainly detecting aMMP‐8 has been developed and substantiated by studies in several countries. (Alassiri et al., 2018; Gul et al., 2020; Sorsa et al., 2016)

One of the recommendations of the 2017/2018 classification is for researchers to develop and validate noninvasive diagnostic tools such as the saliva‐based biomarker diagnostics for the detection of gingival inflammation. (Chapple et al., 2018) In this regard, active aMMP‐8 has thus been implemented as the needed biomarker in the staging and grading of periodontitis (Sorsa et al., 2020) as per the new classification (Table 1). (Tonetti et al., 2018) This is based on decades of consistent and focused research on biomarkers especially, aMMP‐8 in several studies conducted in different parts of the world and among different populations. (Sorsa et al., 1990; Sorsa et al., 2016) The authors have validated aMMP‐8 as a potent and reliable biomarker of periodontal tissue destruction.

**Table 1:** Adjunctive use of active matrix metalloproteinase‐8 (aMMP‐8) as a grading biomarker for a periodontitis patient modified from the recent classification. (Tonetti et al., 2018) The suggested cut‐offs for the levels of aMMP‐8 are based on using aMMP‐8 lateral flow point‐of‐care test among 150 Greek patients as described. (Grigoriadis et al., 2019; Grigoriadis et al., 2021; Sorsa et al., 2020) this table (Sorsa et al., 2020) is reproduced under the terms and conditions of the creative commons attribution (CC BY) license (http://creativecommons.org/licenses/by/4.0/)

Grading a periodontitis patient by aMMP‐8		Grade A: Slow rate of progression	Grade B: Moderate rate of progression	Grade C: Rapid rate of progression
Indicators of active periodontal tissue destruction/bone loss/clinical attachment loss	Mouthrinse, gingival crevicular fluid	No/slow = aMMP‐8 level < 20 ng/ml	Moderate = aMMP‐8 level ≥ 20 ng/ml	Rapid = aMMP‐8 level > 30 ng/ml

It stems from the significant neutrophilic collagenolytic (MMP‐8) activity attributed to aMMP8 in oral fluids such as gingival crevicular fluid such that its elevation associates very closely with disease severity and activity. (Alassiri et al., 2018; Gul et al., 2020; Gupta, Sahni, et al., 2021; Hernández et al., 2021; Sorsa et al., 1990; Sorsa et al., 2016) A direct correlation was also established between poor oral hygiene and periodontal inflammation using an aMMP‐8 lateral flow immunoassay and reported a very high sensitivity (96%) for poor oral hygiene. (Nwhator et al., 2014) Considering the episodic nature of periodontitis, the difficulty in making an accurate assessment of disease progression using clinical measures alone was highlighted. (Herr et al., 2007) To tackle this, the researchers developed and successfully utilized microfluidic immunoassays to rapidly quantify mouthrinse aMMP‐8, and aid in the monitoring of the dynamic activity in periodontitis. (Herr et al., 2007) From their study, the authors asserted the relevance of aMMP‐8 as a biochemical indicator of periodontitis severity and activity to improve and monitor the timing of MMP inhibitor therapy particularly during the active periodontitis phase. This underscores the value of aMMP‐8 as a component of a biomarker panel. (Herr et al., 2007; Sorsa et al., 2020)

## 5 ESTABLISHING A SYSTEMIC LINK VIA THE AMMP‐8 BIOMARKER

In the latest periodontitis definition, it has been suggested that there is the need to identify new approaches to represent aspects of the likely systemic impact of specified periodontitis cases and its therapy. (Tonetti et al., 2018) This detailed review (Beck et al., 2019) sheds more light on this, as they described a plethora of studies on the role of C‐reactive protein (CRP) as pertinent biomarkers of systemic inflammation. One of such studies found elevated CRP as a very good indicator of cardiovascular events in healthy women, and this has now become one of the building blocks for using CRP as a diagnostic tool, in inflammatory diseases, including oral infection. (Beck et al., 2019; Ridker et al., 1998) Emerging evidence from some studies have also reported the impact of periodontal therapy of periodontitis on general health benefits. (Tonetti et al., 2018) This is underpinned by the beneficial effects of nonsurgical periodontal therapy on systemic levels of CRP, and other measures of inflammation leading to a decreased risk of cardiovascular events (Beck et al., 2019; Bokhari et al., 2012) and in participants that were not on antihypertensive medications. (Beck et al., 2019; Zhou et al., 2017) The association between aMMP‐8 and systemic conditions such as obesity has indeed been demonstrated. (Lauhio et al., 2016) To further support the link between MMP‐8 and systemic inflammation, it has been demonstrated that circulating MMP‐8 positively correlate with CRP levels in acute coronary syndrome. (Allal‐Elasmi et al., 2014) It was demonstrated that aMMP‐8 had the ability to proteolytically process and degrade the human insulin receptor in the serum of obese individuals, thus eventually promoting and reflecting insulin resistance and its development. (Lauhio et al., 2016) Doxycycline, an MMP‐8 inhibitor was shown to successfully inhibit MMP‐8. (Emingil et al., 2019; Lauhio et al., 2016) These results indicate that MMP‐8 is an important mediator in the systemic subclinical inflammatory response in obesity, as well as a prospective drug target. (Lauhio et al., 2016) Host modulation therapy through the administration of sub‐antimicrobial doxycycline reduces systemic collagenolytic and pro‐inflammatory (hsCRP, TNF‐α, MMP‐9) biomarkers including MMP‐8 and this therapy have been demonstrated to be an effective adjunct to nonsurgical periodontal treatment in the management of periodontitis. (Emingil et al., 2019; Golub & Lee, 2020) In another randomized, placebo‐controlled, double‐blind trial, 3 months of sub‐antimicrobial dosage of doxycycline adjunctive usage with nonsurgical periodontal treatment significantly reduced some GCF markers of periodontal tissue destruction (MMP‐8, osteoprotegerin and myeloperoxidase) and the clinical periodontal parameters in the subsequent 12‐month period. (Emingil et al., 2019)

Also recently, significant associations were found between aMMP‐8 test and prediabetes after adjusting for BMI and older age. (Grigoriadis et al., 2019) In a similar study, the authors observed that combining periodontitis staging, BMI, increasing age, along with aMMP‐8, served as a viable screening strategy in the dental setting for screening prediabetes/diabetes, without necessarily assessing chairside HbA1c. (Grigoriadis et al., 2021) This may show the ability of prediabetes and diabetes to upregulate aMMP‐8 in inflamed gingiva and oral fluids. (Sorsa et al., 1992) The links between circulating systemic and oral fluid/mouthrinse aMMP‐8 with periodontal findings and the new staging and grading classifications in a dose–dependent manner strongly suggests an association between periodontal disease and enhanced disease activity of diabetes and CVD susceptibility. (Biyikoğlu et al., 2009; D'Aiuto et al., 2018; Kardeşler et al., 2010; Keles Yucel et al., 2020; Noack et al., 2017) Furthermore, GCF aMMP‐8 levels in rheumatoid arthritis (RA) patients and systemically healthy subjects suggest that RA may create a tendency to overproduce this MMP. In a recent pilot study, (Keskin et al., 2021) the authors demonstrated a correlation between pro‐inflammatory biomarkers (aMMP‐8 and Interleukin‐6) and clinical periodontal parameters (rapid progression of clinical attachment loss, increasing probing depths and BOP) in head and neck cancer (HNC) patients after 1 month of radiotherapy. This rapid advancement was from grade A to grade C of periodontitis based on the new system of classification. (Tonetti et al., 2018) The authors therefore designated aMMP‐8 and interleukin‐6 as potentially useful diagnostic tools in monitoring the development and effects of the degenerative deterioration of the oral immunity induced by radiotherapy of HNC. (Keskin et al., 2021)

The close link between serum MMP‐8 levels and systemic inflammatory markers, suggests a physiological relationship between them. (Sirniö et al., 2018) The tumor expression of MMP‐8 has also been demonstrated to positively correlate with cancer progression in ovarian cancer, (Stadlmann et al., 2003) and worse overall survival in hepatocellular carcinoma. (Lempinen et al., 2013) Recently, high‐serum MMP‐8 levels were associated with systemic inflammation and adverse outcome in colorectal cancer (CRC) and it was suggested that serum MMP‐8 could therefore be a pertinent additional prognostic parameter in CRC. (Sirniö et al., 2018) Paradoxically, some studies have reported a possible protective role of MMP‐8 in the initiation and progression of cancer implying that MMP‐8 could be oncosuppressive in various cancers. (Decock et al., 2008; Decock et al., 2011; Korpi et al., 2008) For instance, in breast cancer, elevated plasma levels of MMP‐8 seem to have a protective effect against lymph node metastasis, (Åström et al., 2017) and squamous cell carcinoma of the tongue, in which high‐tumor MMP‐8 expression had associations with improved cancer‐specific survival. (Decock et al., 2008) This briefly highlights the potential double‐sided effects of MMP‐8 in the oral‐systemic link, which calls for further investigation.

Just recently, it was hypothesized that aMMP‐8 could be useful in assessing the risk of deterioration and complications in COVID‐19 patients if a causality is established between periodontal disease and severe COVID‐19 infections. (Räisänen et al., 2020) Recent studies have revealed by clinical assessments and aMMP‐8 POC testing that periodontal disease can thus be regarded as a possible risk disease for COVID‐19 infection. (Gupta, Mohindra, et al., 2021; Sorsa et al., 2021) This could be beneficial in screening and identifying such patients in places where professional dental expertise and equipment are limited or unavailable such as nursing homes, care homes, rural and distant locations. (Räisänen et al., 2020) Following the hypothesis, (Räisänen et al., 2020) evidence has emerged implying periodontitis as a possible risk factor for a higher risk of ICU admissions, the need for assisted ventilation and death of COVID‐19 patients in Qatar and India, notably with elevated blood levels of biomarkers (white blood cell levels, D‐dimer, and CRP) linked to worse disease outcomes. (Gupta, Mohindra, et al., 2021; Marouf et al., 2021; Sorsa et al., 2021)

Thus, it is logical to reason that the inclusion of validated biomarkers (Tonetti et al., 2018) would improve diagnostic accuracy in the timely detection of periodontitis and give a more precise and eventually predictive assessment of periodontitis grade. (Herr et al., 2007; Lee et al., 1995; Leppilahti et al., 2015; Sorsa et al., 2020)

## 6 CONCLUSIONS

The usefulness and versatility of aMMP‐8 is invaluable as a grading biomarker for periodontitis as well as an important measure of the oral/periodontal systemic link; that is, mouth‐body connection. We therefore propose the use of validated, simple mouthrinse/oral fluid aMMP‐8 POCT assay. (Herr et al., 2007; Nwhator et al., 2014; Sorsa et al., 2016; Sorsa et al., 2020) as a more sensitive marker for systemic diseases such as subclinical atherosclerosis, diabetes and obesity (Grigoriadis et al., 2019; Grigoriadis et al., 2021; Lauhio et al., 2016) and propose/suggest the inclusion of aMMP‐8 as an indicator of grading of the clinical attachment loss/bone loss or collagen destruction. In this regard, the utility of aMMP‐8 needs further interdisciplinary validations.

## CONFLICT OF INTEREST

Prof Timo Sorsa is the inventor of 1,274,416‐patent, U.S. 5,652,223, 5,736,341, 5,864,632, 6,143,476 and US 2017/0023571A1 (issued June 6, 2019), WO 2018/060553 A1 (issued May 31, 2018), 10,488,415 B2, and US 2017/0023671A1, Japanese Patent 2016‐554676 and South Korean Patent No. 10‐2016‐7025378. Ismo T. Räisänen has received dissertation grants from The Yrjö Jahnsson Foundation sr, The Paulo Foundation, The Emil Aaltonen Foundation sr, The Juhani Aho Foundation for Medical Research sr, The Orion Research Foundation sr, The Finnish Dental Society Apollonia and Helsingin Seudun Hammaslääkärit r.y. (Dentists of Helsinki Region Association), Finland. Other authors report no conflicts of interest related to this study. The funders had no role in the study design; the collection, analyses, or interpretation of the data; the drafting of the manuscript, or in the decision to publish the results.

## AUTHOR CONTRIBUTIONS

Kehinde Adesola Umeizudike contributed to conception, design, data acquisition, analysis, and interpretation, drafted and critically revised the manuscript; Ismo Räisänen contributed to data acquisition, analysis, and interpretation, drafted and critically revised the manuscript; Shipra Gupta contributed to conception, design, data analysis, and interpretation, drafted and critically revised the manuscript; Solomon Nwhator contributed to data acquisition, and interpretation, critically revised the manuscript; Andreas Grigoriadis contributed to conception, design, data acquisition, analysis, and interpretation, critically revised the manuscript; Dimitra Sakellari contributed to data acquisition, analysis, and interpretation, critically revised the manuscript; Timo Sorsa contributed to conception, design, data acquisition, analysis, and interpretation, drafted and critically revised the manuscript. All authors gave their final approval and agreed to be accountable for all aspects of the work.

## Data Availability

Data sharing not applicable ‐ no new data generated
